# Effect of Airborne Particulate Matter on the Immunologic Characteristics of Chronic Rhinosinusitis with Nasal Polyps

**DOI:** 10.3390/ijms23031018

**Published:** 2022-01-18

**Authors:** Hyun-Joo Lee, Dong-Kyu Kim

**Affiliations:** 1 Institute of New Frontier Research, Hallym University College of Medicine, Chuncheon 24253, Korea; leekul79@gmail.com; 2 Department of Otorhinolaryngology-Head and Neck Surgery, Chuncheon Sacred Heart Hospital, Hallym University College of Medicine, Chuncheon 24253, Korea

**Keywords:** rhinosinusitis, nasal polyp, particulate matter, Th2, interleukin-33, ST2

## Abstract

The inflammatory mechanisms of environmental pollutants in chronic rhinosinusitis (CRS) have recently been proposed. However, the mechanisms underlying the inflammatory effects of particulate matter (PM) on nasal polyp (NP) tissues remain unknown. Here we investigated the mechanism underlying the inflammatory effects of PM10 on human nasal polyp-derived fibroblasts (NPDFs). We isolated NPDFs from human NP tissues obtained from patients with CRS with NPs (CRSwNP). The NPDFs were exposed to PM10 in vitro. Immunologic characteristics were assessed using real-time polymerase chain reaction, enzyme-linked immunosorbent assay, Western blot, and flow cytometry. Additionally, we investigated the effect of NPDF-conditioned media (CM) on the expression of CD4^+^ T cell inflammatory mediators. PM10-treated NPDFs significantly upregulated interleukin (IL)-6, IL-4, and IL-33 expression and CXCL1 protein levels than PM10-treated normal tissues. MAP kinase, AP-1, and NF-kB were the primary cell signaling proteins. Immune cells in NPDF-CM had elevated IL-13, IL-17A, and IL-10 expression, but no significant difference in IFN-γ, TNF-α, and IL-4 expression. Moreover, under a Th2 inducing condition, NPDF-CM-treated CD4^+^ T cells had increased expression of IL-13, IL-10, and IL-17, which was reversed on ST2 inhibitor addition. Our study suggests that PM10 exposure could significantly increase the Th2 inflammatory pathway in NP tissues, specifically the IL-33/ST2 pathway-mediated immune response.

## 1. Introduction

Chronic rhinosinusitis (CRS) is a common otorhinolaryngologic disease characterized by nasal and paranasal sinus mucosa inflammation with or without accompanying nasal polyp (NP) formation. Histologically, CRS features significant goblet hyperplasia, mucus hypersecretion, mucociliary dysfunction, and persistent sinonasal inflammation. Epidemiologic studies showed that it affects approximately 10–15% of the population and substantially impairs patients’ quality of life [1,2,3]. Although CRS is a multifactorial disease, several studies have reported that air pollution, such as particulate matter (PM), is a principal environmental risk factor for CRS [4,5,6]. Additionally, increased fine particulate exposure has been associated with the development of sinusitis among adults [7].

Due to rapid industrialization and urbanization, air pollution has become one of the main risk factors for disease. PM generally refers to solid and liquid particles suspended in ambient air. Generally, PM is classified according to diameter into PM10 (particles smaller than 10 μm) and PM2.5 (particles smaller than 2.5 μm). Due to its small size, PM2.5 is known to damage the lower respiratory tract. The effects of PM on the inflammatory changes of the lower airway have been well characterized, including alterations in gene transcription, cytokine production, and macrophage polarization [8,9,10]. These inflammatory changes also involve the direct and indirect production of reactive oxygen species, which damage human cellular components such as DNA, lipids, and proteins, ultimately leading to downstream inflammatory changes in the affected organs [11,12].

In contrasting, the impact on upper airway disease has not been clearly defined, specifically in relation to PM10. A recent epidemiologic study in South Korea showed that PM10 is associated with an increased risk of CRS [13]. However, studies investigating how PM10-induced inflammatory changes affect patients with CRS with NPs (CRSwNP) are lacking. Therefore, the aim of this study was to explore the potential pathogenic role of PM10 in patients with CRSwNP using tissues from human nasal polyp-derived fibroblasts (NPDFs).

## 2. Results

### 2.1. Increased Expression of IL-6, IL-4, IL-33, and CXCL1 on PM10-Treated Human NPDFs

**Figure 1:**
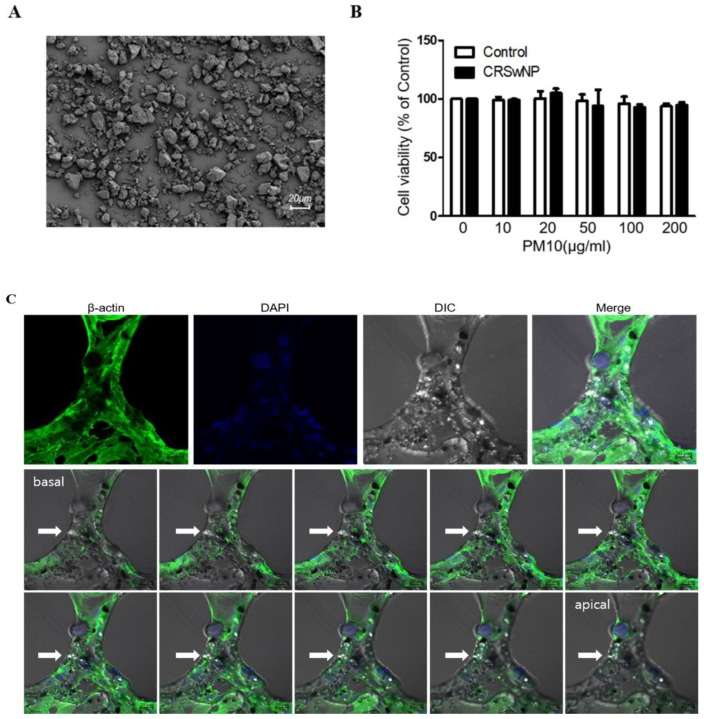
The effect of PM10 on human nasal polyp derived fibroblasts (NPDFs). (**A**) Representative image of PM10 using scanning electron microscopy. (**B**) PM10 did not affect the viability of NPDFs until the concentration reached 200 μg/mL. The graphic data represents the means ± SEM of three independent experiments. (**C**) Z-stack confocal immunofluorescence analysis of PM10 treated-NPDFs. Cells were stained with β-actin (green) and DAPI stains cell nuclei. Arrows indicate PM10, Bar = 20 μm. DIC: Differential interference contrast microscopy.

Figure 1A shows the images of the ERM-CZ100 (PM10) used in this study. The particles of PM10 showed various sizes under 20 µm in diameter. These were certified to include seven polycyclic aromatic hydrocarbons (PAHs), having the highest amount of benzo [b] fluoranthene and also contained benzo[a]anthracene, benzo[k]fluoranthene, benzo[j] fluoranthene, benzo[a]pyrene, indeno [1,2,3-c,d] pyrene, and dibenzo[a,h]anthracene. The CCK-8 assay indicated that exposure of nasal tissues to different concentrations of PM10 (0–200 μg/mL) for 24 h had no significant effect on viability (Figure 1B). To identify the detection of cellular uptake of PM10, we performed Z-stack confocal immunofluorescence analysis (Figure 1C). We found that the particles of PM10 were well-internalized in the NPDFs.

**Figure 2:**
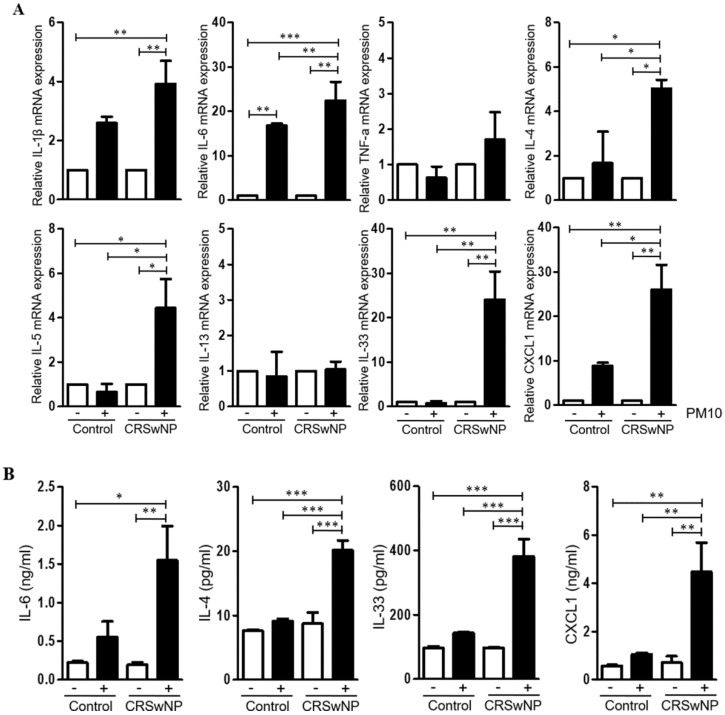
Cytokine and chemokine expression in human nasal polyp derived fibroblasts (NPDFs) after PM10 exposure. (**A**) The mRNA expression level of various cytokines and chemokines in NPDFs treated with PM10 (200 μg/mL) for 24 h. PM10-treated NPDFs had higher IL-6, IL-4, IL-33, and CXCL1 expression than untreated NPDFs or normal nasal tissue treated with PM10. (**B**) The protein expression levels of IL-6, IL-4, IL-33, and CXCL1 were increased in PM10-treated NPDFs. The graphic data represents the means ± SEM (*n* = 5). * *p* < 0.05; ** *p* < 0.01, *** *p* < 0.0001.

Next, to investigate the immunologic effect of PM10 on NPDFs, we evaluated the expression level of various cytokines and chemokines in PM10-treated NPDFs using qPCR for each PM10 concentration (Figure 2A). We found that the level of IL-6, IL-4, IL-5, IL-33, and CXCL1 mRNA expression was significantly higher in NPDFs treated with PM10 than in untreated or normal nasal tissue treated with PM10. Additionally, we detected higher IL-6, IL-4, IL-33, and CXCL1 protein levels on PM10-treated NPDFs (Figure 2B).

### 2.2. Cellular Signaling Pathway Elicited by PM10 in Human NPDFs

**Figure 3:**
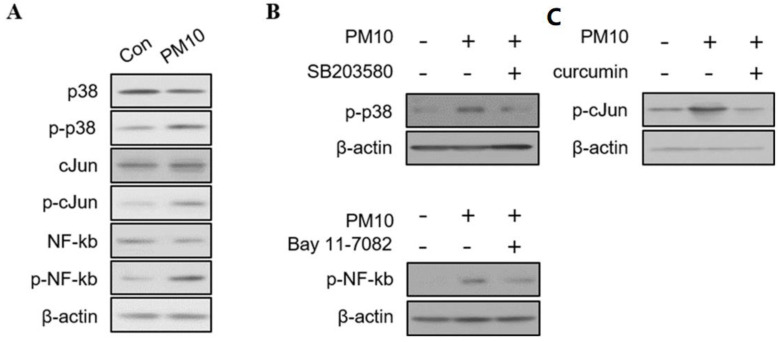
The immunoblotting results of signaling pathways in human nasal polyp derived fibroblast (NPDFs) after exposure to PM10. (**A**) Western blots of cytoplasmic proteins with antibodies to MAPKs (p38), AP-1 (c-Jun), and NF-kB in PM10 (200 μg/mL)-treated NPDFs for 1 h. (**B**) The ratio of phosphorylation of p38, c-Jun, and NF-kB by adding their respective inhibitors (SB203580 for p38, curcumin for c-Jun, bay 11-7082 for NF-kB). β-actin was used as an internal control. The phosphorylated protein band values were normalized to total protein bands. (**C**) The mRNA expression and protein levels of IL-6, IL-4, IL-33, and CXCL-1 were significantly inhibited by the addition of inhibitors to PM10-treated NPDFs. Relative intensities of blot bands were measured with Image J software. The graphic data represents the means ± SEM (*n* = 5).

To determine the cellular signaling pathways elicited by PM10 exposure, we assessed the levels of phosphorylated MAP kinase (p38), AP-1 (c-Jun), and NF-kB. The Western blot revealed increased phosphorylation of p38, c-Jun, and NF-kB according to the concentration of PM10 (Figure 3A). Additionally, the ratio of phosphorylation of p38, c-Jun, and NF-kB was reversed by the addition of their respective inhibitors (SB203580 for p38 inhibitor, curcumin for c-Jun inhibitor, bay 11-7082 for NF-kB inhibitor) (Figure 3B).

**Figure 4:**
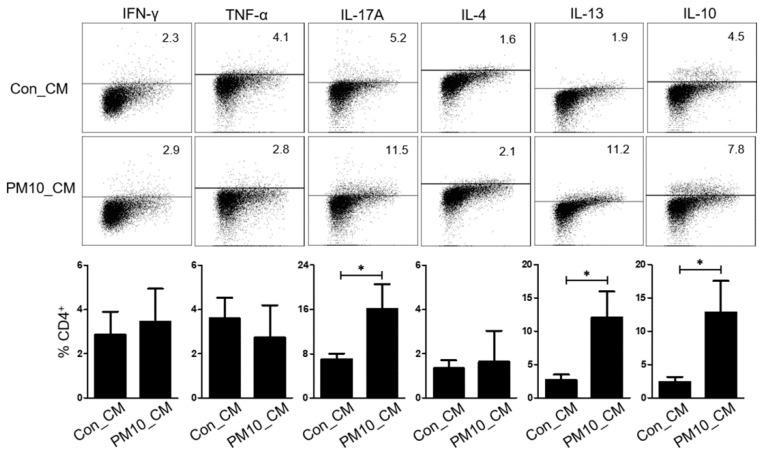
Effect of human nasal polyp derived fibroblast-conditioned media (NPDF-CM) treated with PM10. The graphic data represents the means ± SEM (*n* = 3). * *p* < 0.05.

We also investigated the immunological cytokine profile by exposing PM10-treated NDPFs to each inhibitor (Figure 3C). The addition of the inhibitors to PM10-treated NPDFs significantly reduced the mRNA expression of IL-6, IL-4, IL-33, and CXCL1 (Figure 4). The protein levels of IL-6, IL-4, IL-33, and CXCL-1 were also down-regulated by the signaling pathway inhibitors, except the inhibition of CXCL1 expression by bay 11-7082 (Figure 3).

### 2.3. Effect of PM10-Treated Human NPDFs on Cytokine Expression of CD4^+^ T Cells

First, we explored whether the NPDF-conditioned media (NPDF-CM) could affect cytokine expression in immune cells. Tonsil-derived mononuclear cells (TMCs) incubated in NPDF-CM for 72 h, had increased expression of IL-13, IL-17A and IL-10 compared to those not incubated in NPDF-CM (Figure 4). However, the immune cells did not differ significantly in the expression of IFN-γ, TNF-α, and IL-4.

**Figure 5:**
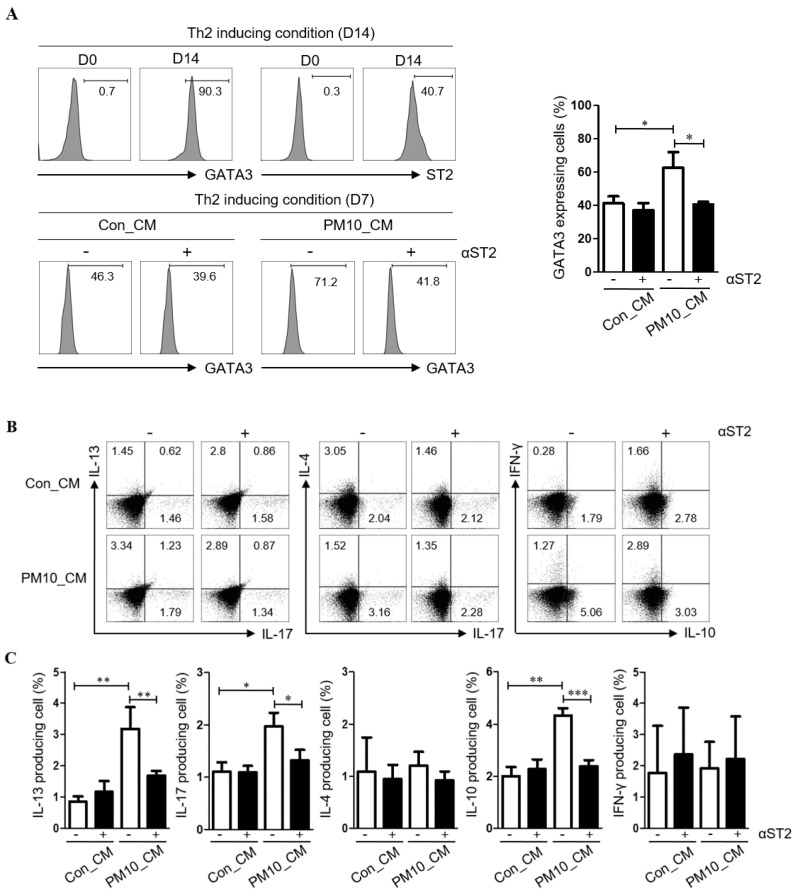
The pivotal effect of PM10-induced IL-33 for Th2 differentiation effects of conditioned media on PM10-induced human nasal polyp derived fibroblast (NPDFs-CM) on the inflammatory cytokine production in CD4^+^ T cells. (**A**) Th2 differentiation was determined by the expression of GATA3 and ST2 (IL-33 receptor). (**B**,**C**) The protein levels of IFN-γ, IL-17A, IL-4, IL-13, and IL-10 in CD4^+^ T cell populations were determined by flow cytometry according to the addition of ST2. The graphic data represents the means ± SEM (*n* = 3). * *p* < 0.05, ** *p* < 0.01, *** *p* < 0.0001 as compared to the PM10-treated group.

To examine the effect of PM10-induced IL-33 on Th2 differentiation, CD4^+^ T cells were cultured in NPDF-CM under Th2 inducing conditions (Figure 5A), and Th2 differentiation was determined by the expression of GATA3 and ST2 (IL-33 receptor). Flow cytometric analysis revealed that the levels of GATA3 were increased in CD4^+^ T cells treated with NPDF-CM under in vitro Th2-inducing conditions (Figure 5A). Additionally, NPDF-CM-treated CD4^+^ T cells had high levels of IL-13, IL-10, and IL-17, whereas ST2 neutralization reversed the expression of IL-13, IL-10, and IL-17 (Figure 5B,C).

## 3. Discussion

To date, few studies have investigated the underlying mechanism of the effects of PM10 on the inflammatory changes of the upper airway, specifically NP tissues. In this study, we assessed the underlying mechanism of PM10-induced inflammatory changes in human NPDFs. We found that PM10 effectively mediated inflammatory changes on NPDFs via MAP kinase, AP-1, and NF-kB by increasing the expression of IL-6, IL-4, IL-33, and CXCL1. We speculated that the inflammatory changes induced in the NPDFs could increase the expression of IL-13, IL-17A, IL-10 in immune cells. Indeed, when CD4^+^ T cells were cultured in NPDF-CM, GATA3 and ST2 (markers for Th2 induction) were increased. Moreover, CD4^+^ T cells treated with NPDF-CM significantly upregulated IL-13, IL-10, and IL-17 expression, whereas ST2 neutralization inhibited those expressions in CD4^+^ T cells.

It has long been noted that PM can produce an inflammatory response in airways. PM has been shown to induce the expression of several pro-inflammatory cytokines, including TNF-α, IFN-γ, IL-1, IL-6, IL-8, and IL17A by neutrophils, macrophages, and epithelial cells in the airway [14,15,16]. One recent study also revealed that IL-32 induced by Asian sand dust is known to play an important role in the late stages of chronic inflammatory airway diseases [17]. Additionally, several studies have reported that PM impairs tight-junction integrity at a cellular level [18,19,20]. Thus, these events may lead to sinonasal disease caused by PM. However, it remains unclear whether PM can predispose individuals to nasal polypogenesis and lead to disease exacerbation or progression, or if it contributes to the persistence of an existing disease process.

Epithelial cells are the first physiological barrier against invasion by pathogens and allergens, and dysfunction of the epithelial barrier is associated with allergic diseases. Previous studies have indicated that co-exposure to an allergen and PM could enhance Th2 responses with upregulated IgE production and eosinophilia [21,22]. A previous study showed that PM2.5 exposures were associated with an increased nasal percentage of eosinophils and alpha 1-antitrypsin concentrations in children with asthma children but not in healthy children [23]. An animal study showed that PM co-exposure with house dust mites resulted in airway hyperresponsiveness and increased Th2 cytokine levels in mice [24]. In line with our data, one previous study showed that diesel exhaust particles induce the upregulated expression of IL-6 and IL-8 levels via p38, Akt, and NF-κB signaling pathways in nasal fibroblasts [25]. Another study showed that Asian sand dust induces the increased IL-6 and IL-8 expression via the MAPK/CREB signaling pathways in nasal fibroblasts [26]. One recent study also insisted that PM can influence neutrophils, eosinophils, macrophages, or fibroblasts, particularly if the epithelial-to-mesenchymal transition takes place [27,28]. Collectively, these studies suggest that PM could contribute to developing nasal polypogenesis.

Moreover, PM-exposed mice have also been shown to have elevated expression of Th2 inflammatory mediators such as IL-13 and eotaxin-1, as well as decreased expression of IFN-γ and IL-12p40 [29]. In the present study, we newly found that PM10 exposure increases type 2 inflammatory responses through MAP kinase, AP-1, and NF-kB signaling pathways. Moreover, NPDFs-CM co-cultured with PM10 contributed to Th2 induction in CD4^+^ T cells, which resulted in the increased production of IL-13, IL-17A and IL-10. Recently, several studies showed that epithelium-derived cytokines, namely IL-25, IL-33, and TSLP, act as key regulatory factors in immune-pathogenic mechanisms of allergic rhinitis, asthma, and CRSwNP mainly involved in type 2 inflammatory responses [30]. Specifically, IL33 is a key cytokine involved in type 2 immunity and allergic airway diseases. It interacts with dendritic cells, Th2 cells, follicular T cells, and regulatory T cells; thus, it plays critical roles in the development of chronic airway inflammation and tissue remodeling [31]. Consistent with this, we found that IL-33 plays an important role in Th2 induction of CD4^+^ T cells when exposed to NPDFs-CM co-cultured with PM10. Contrary to our findings, a recent study demonstrated that exposure of cultured nasal epithelial cells to PM2.5 did not affect the expression of epithelium-derived cytokines, such as IL-25 and IL-33 [32]. We speculate that this discrepancy may be due to the use of different tissues (normal nasal tissues and NPDFs) and different types of PM (PM2.5 and PM10). However, this study also demonstrated the loss of barrier function and the increased production of TSLP as another epithelium-derived cytokine involved in type 2 immunity.

In conclusion, PM10 may play an important role in eliciting inflammatory changes in NP tissues via MAP kinase, AP-1, and NF-kB, indicating that PM10 can contribute to the increased expression of IL-6, IL-4, IL-33, and CXCL1. Additionally, it may provoke the upregulation of IL-13, IL-17A, IL-10 in immune cells and induce the Th2 receptor of CD4^+^ T cells via the IL-33/ST2 pathway. Therefore, PM10 could lead to disease exacerbation or progression in patients with CRSwNP.

## 4. Materials and Methods

### 4.1. Isolation of Human NPDF

Samples of NP tissues were obtained from five patients with CRSwNP during routine endoscopic sinus surgery. For each patient, the sinus disease diagnosis was based on patient history, clinical examination, nasal endoscopy, and computed tomography of the paranasal sinuses, as detailed by the guidelines contained in EPOS 2020: European position paper on rhinosinusitis and NPs 2020 [33]. Patients who used oral or nasal corticosteroids or antibiotics for 4 weeks before sample collection were excluded from the study. The tissue specimens were enzymatically digested using collagenase type I (Worthington Biochemical Corporation, Lakewood, NJ, USA) and Dnase (Sigma-Aldrich, St. Louis, MO, USA for 30 min at 37 °C under stirring and placed in Dulbecco’s modified Eagle’s medium-high glucose (Welgene Inc., Gyeongsan, Korea) supplemented with 10% fetal bovine serum (Gibco, Grand Island, NY, USA), 100 U/mL penicillin (Sigma-Aldrich), and 100 mg/mL streptomycin (Sigma-Aldrich). After 2 days, floating cells were removed by changing the medium. To confirm whether the cultured spindle-shaped cells were nasal fibroblasts, the cells were analyzed as previously described [34]. To prepare conditioned media (CM), the confirmed nasal fibroblasts (1 × 10^5^ cells/well) were seeded into 6-well tissue culture plates and treated with PM10 (200 μg/mL, ERM certified reference material ERM-CZ100 (PM10-like), Sigma-Aldrich) at 37 °C for 24 h. After 24 h, the CM was harvested, centrifuged at 13,000 rpm for 10 min, filtered using a 0.22 μm syringe filter, aliquotted, and stored at −80 °C until required.

### 4.2. Scanning Electron Microscopy

PM10 was mounted on double-slide carbon tape and sputter-coated with gold in an ion sputter coater (E-1010, Hitachi, Japan). The morphology of PM10 particles was observed under a scanning electron microscope (Analytical HR- SEM; Carl-Zeiss, Jena, Germany) in Chuncheon Center of Korea Basic Science Institute. The instrument was operated at 3.0 kV.

### 4.3. Z-Stack Confocal Immunofluorescence Analysis

First, NPDFs were stimulated with 200 μg/mL of PM10 for 24 h. Nonadherent PM10 was harvested by simply pipetting. Next, the particles were washed three times to remove nonadherent particles. Adherent particles were detached by thorough and careful pipetting so as not to detach plastic-adherent NPDFs. The NPDFs were fixed with cold methanol, blocked with 5% normal goat serum, and stained with primary antibody against β-actin antibody (Cell signaling Technology, Danvers, MA, USA) overnight at 4 °C and incubated with Alexa 488 conjugated secondary antibody (Invitrogen, Waltham, MA, USA) for 1 h. After mounting with DAPI, Z-stack confocal immunofluorescence analysis was performed by confocal microscopy (Carl Zeiss, Germany).

### 4.4. Quantitative Real-Time PCR

To evaluate the mRNA expressions of cytokines and chemokines in nasal fibroblasts after treatment with PM10, the nasal fibroblasts were exposed to PM10 (200 μg/mL) for 24 h. Total RNA was isolated using an easy-BLUE reagent (Intron Biotechnology, Seongnam, South Korea) according to the manufacturer’s recommendations. cDNAs were synthesized from 2 μg of total RNA using the AccuPower cDNA synthesis kit (Bioneer, Daejeon, Korea). The prepared cDNA was amplified and quantified using the SYBR Green master mix (Applied Biosystems, Foster City, CA, USA) with the following primers: β-actin, forward 5′-GTG CTA TCC CTG TAC GCC TC-3′ and reverse 5′-GGC CAT CTC TTG CTC GAA GT-3′; IL-1β, forward 5′-ATG ATG GCT TAT TAC AGT GGC AA-3′ and reverse 5′-GTC GGA GAT TCG TAG CTG GA-3′; IL-6, forward 5′-AAT TCG GTA CAT CCT CGA CGG-3′ and reverse 5′-GGT TGT TTT CTG CCA GTG CC-3′; TNF-α, forward 5′- TGT AGC CCA TGT TGT AGC AAA CC-3′ and reverse 5′-GAG GAC CTG GGA GTA GAT GAG GTA- 3′; IL-4, forward 5′-CCG AGT TGA CCG TAA CAG ACA T-3′ and reverse 5′-GTC CTT CTC ATG GTG GCT GTA G-3′; IL-5, forward 5′-CAG GGA ATA GGC ACA CTG GAG-3′ and reverse 5′-GCA CAG CCA GGA CAA ATA TAG C-3′; IL-13, forward 5′-CAA GGT CTC AGC TGG GGT AA-3′ and reverse 5′-GGA TAT TCA GCC AGC TTC CCT T-3′; IL-33, forward 5′-ACA GAA TAC TGA AAA ATG AAG CC-3′ and reverse 5′CTT CTC CAG TGG TAG CAT TTG-3′; CXCL1, forward 5′-GCG CCC AAA CCG AAG TCA TA-3′ and reverse 5′-ATG GGG GAT GCA GGA TTG AG-3′. Polymerase chain reactions were performed using a real-time thermal cycler system (Qiagen, Hilden, Germany).

### 4.5. Western Blot Analysis

To determine the signaling pathways elicited by PM10, NPDFs were pretreated with the following signaling pathway inhibitors: SB203580 (p38 inhibitor, 10 μmol/L; Sigma-Aldrich), C-Jun (curcumin, 10 μmol/L; Sigma-Aldrich) or BAY117082 (NF-kB inhibitor, 2.5 μmol/L; Sigma-Aldrich). After a 1 h treatment of the inhibitors, the cells were incubated with PM10 (200 μg/mL) for 6 h and then lysed in LIPA buffer. Lysates were separated using 10% sodium dodecyl sulfate-polyacrylamide gel electrophoresis and transferred onto polyvinylidene fluoride membranes (Millipore, Billerica, MA, USA). The membranes were blocked with blocking solution and incubated with antibodies to NF-kb (Cell signaling Technology, Danvers, MA, USA), p38 (Cell signaling Technology), c-Jun (Cell signaling Technology), phospho-NF-kb p65 (Cell signaling Technology), phospho-p38 (Cell signaling Technology), phospho-c-Jun (Cell signaling Technology), and β- actin. Blots were visualized using horseradish peroxide-conjugated secondary antibodies and an Enhanced chemiluminescence (ECL) system (Pierce, Rockford, IL, USA).

### 4.6. Enzyme-Linked Immunosorbent Assay (ELISA)

The levels of IL-6, IL-4, IL-33, and CXCL1 in nasal fibroblasts after treatment with PM10 were detected by ELISA. Nasal fibroblasts were exposed to a concentration of PM10 (200 μg/mL) for 24 h. The cultured cells were lysed in RIPA buffer, and cell lysates were collected to measure protein production. The levels of IL-6, IL-4, IL-33, and CXCL1 were measured using an ELISA kit (Solarbio Science & Technology, Beijing, China) and analyzed using a microplate reader (GloMax^®^ Discover Microplate Reader, Madison, WI, USA). The ELISA procedure was performed following the manufacturer’s recommendations.

### 4.7. Flow Cytometry, Tonsillar Mononuclear Cells, and CD4^+^ T Cells

Tonsils obtained by tonsillectomy were manually cut into small pieces and exposed to the enzymes, DNase I, and collagenase type I (Sigma-Aldrich) for 30 min at 37 °C under stirring [35]. This solution was then filtered through a 70 μm cell strainer to collect single-cell suspensions. Tonsillar mononuclear cells (TMCs) were obtained using Ficoll-Paque (GE Healthcare, Little Chalfont, United Kingdom) density gradient centrifugation. TMCs (1 × 10^6^ cells) were cultured with 1 μg/mL of phytohemagglutinin (PHA) in NPDF-CM for 72 h. For Th2 differentiation, CD4^+^ T cells from TMCs were purified using a CD4^+^ T Cell Isolation Kit MicroBeads (Miltenyi Biotech, Bisley, Surrey, UK), and purified CD4^+^ T cells were differentiated using a CellXVivo Human Th2 Cell Differentiation Kit (RnDSystems, Minneapolis, MN, USA) following the manufacturer’s instructions. In some experiments, 100 ng/mL of ST2 neutralization antibody (eBioscience, CA, USA) was added. The phenotypes of induced Th2 T cells were analyzed by flow cytometry. Cytokine production was confirmed after restimulating cells with phorbol 12-myristate 13-acetate (40 ng/mL; Sigma-Aldrich) and Ionomycin (1 μg/mL; Sigma-Aldrich) for 5 h. To block exocytosis of cytokines, cells were treated with Protein Transport Inhibitor Cocktail (eBioscience) for the last 4 h. Cell surfaces were stained with fluorescein-conjugated CD4 (eBioscience) for 20 min at 4°C in the dark. After two washings with wash buffer, we performed intracellular staining for cytokine production and GATA3 detection. For that purpose, the cells were fixed and permeabilized using Foxp3/Transcription Factor Staining Buffer Set kit (eBiosciences) and then stained with Phycoerythrin (PE) anti-IL-13 (Biolegend, San Diego, CA, USA), PE-cy7 conjugated anti-IFN-g and anti-IL-4 (Biolegend), allophycocyanin (APC) conjugated anti-IL-17 (Biolegend), APC conjugated anti-IL-10 (Biolegend), APC-cy7 conjugated anti-TNF-α (Biolegend), and stained with Peridinin-Chlorophyll-Protein (PerCP) cy5.5 conjugated GATA3 (eBiosciences). The stained cells were analyzed using FACSCantoⅡ (Temecula, CA, USA). Acquired data were analyzed using FlowJo software (TreeStar Inc., Ashland, OR, USA).

### 4.8. Statistical Analysis

All experiments were performed in triplicate. The data are expressed as the mean ± standard error (SEM). Statistically significant differences between control and experimental data were analyzed using an unpaired Student’s t-test or one-way analysis of variance (ANOVA) followed by Tukey’s test (GraphPad Prism, version 5, GraphPad Software, CA, USA).

## Data Availability

The authors confirm that the data supporting the findings of this study are available within the article.
